# Emergency department visits as a potential opportunity to promote primary care attachment and modify utilization patterns – results of a pilot study in Berlin, Germany

**DOI:** 10.1186/s12873-024-01056-0

**Published:** 2024-08-07

**Authors:** Felix Holzinger, Lisa Kümpel, Rebecca Resendiz Cantu, Anja Alberter, Martin Möckel, Christoph Heintze

**Affiliations:** 1 Charité – Universitätsmedizin Berlin, corporate member of Freie Universität Berlin and Humboldt-Universität zu Berlin, Institute of General Practice, Charitéplatz 1, 10117 Berlin, Germany; 2 Charité – Universitätsmedizin Berlin, corporate member of Freie Universität Berlin and Humboldt-Universität zu Berlin, Division of Emergency Medicine, Campus Mitte and Virchow, Augustenburger Platz 1, 13353 Berlin, Germany

**Keywords:** Emergency department, Primary care attachment, Pilot intervention, Follow-up, Health care utilization

## Abstract

**Background:**

Utilization by low acuity patients contributes to emergency department (ED) crowding. Both knowledge deficits about adequate care levels and access barriers in primary care are important promoters of such presentations. Concurrently, not having a general practitioner (GP) increases the likelihood of low-acuity ED utilization. This pilot study thus investigated feasibility, acceptance, and potential effects of an ED-delivered intervention for low-acuity patients with no regular primary care provider, consisting of an educational leaflet on acute care options and an optional GP appointment scheduling service.

**Methods:**

Low-acuity ED consulters not attached to a GP were given an information leaflet about alternative care offers for acute health problems and offered optional personal appointment scheduling at a local GP practice. Patients were surveyed on demographics, medical characteristics, health care utilization, valuation of the intervention, and reasons for not being attached to a GP and visiting the ED. A follow-up survey was conducted after twelve months. Trends in health and health care utilization were evaluated.

**Results:**

Between December 2020 and April 2022, *n* = 160 patients were enrolled, *n* = 114 were followed up. The study population was characterized by young age (mean 30.6 years) and predominantly good general health. Besides good health, personal mobility was a central reason for not being attached to a GP, but general preference for specialists and bad experiences with primary care were also mentioned. Most frequently stated motives for the ED consultation were subjective distress and anxiety, a belief in the superiority of the hospital, and access problems in primary care. The interventional offers were favorably valued, 52.5% (*n* = 84) accepted the GP appointment scheduling service offer. At follow-up, GP utilization had significantly increased, while there were no significant changes regarding utilization of other providers, including ED. An additional practice survey showed a 63.0% take-up rate for the appointment service.

**Conclusions:**

With this pilot study, we were able to show that a personalized appointment scheduling service seems to be a promising approach to promote GP attachment and increase primary care utilization in patients without a regular GP in a highly urbanized setting. Further larger-scale studies are needed to investigate potential quantitative effects on ED visits.

**Trial Registration:**

German Clinical Trials Register (DRKS00023480); date 2020/11/27.

**Supplementary Information:**

The online version contains supplementary material available at 10.1186/s12873-024-01056-0.

## Background

Emergency department (ED) crowding is a widespread problem, with utilization by low-acuity patients frequently discussed as a contributing factor [1]. Access problems in primary care (PC) are an important determinant of such presentations [2–5]. Concurrently, patients without general practitioner (GP) attachment have been thematized as a group presenting to ED more frequently for minor complaints, while PC integration and care continuity have been described as determinants of apposite utilization patterns [6, 7].

In Germany, there is no obligation to register with a GP, and patients can access both PC providers and EDs freely without any gatekeeping or penalty payments. Data from previous work by our research group in the ED setting suggest that about 15% of patients identify themselves as unattached to a GP practice [8], which slightly exceeds the share of ~ 10% reported from general population surveys [9].

Lack of knowledge about adequate care levels for different types of complaints is another important potential promoter of ED consultations for health issues that could be addressed in PC [10]. Deficits in navigating the complexities of the health care system and choosing the right provider for a particular problem may also contribute to patients not attaching to a PC doctor.

Therefore, the EMAPREPARE intervention for low-acuity ED patients without current attachment to a GP practice was designed to (1) improve knowledge about care options for acute complaints by an information leaflet handed out during the ED visit, and (2) promote individual PC attachment by offering an optional appointment scheduling service. The project was designed as a pilot study, primarily to determine feasibility and acceptance. Results of the qualitative evaluation of the intervention components have already been published [11], and interventions were well received. However, to gain insights into the potential effectiveness in initiating PC attachment and a potential impact on consultation patterns, the quantitative prospective part of the study included a comprehensive follow-up (FU) module. This paper reports on the quantitative results of the EMAPREPARE pilot intervention study, including longitudinal trends.

## Methods

### Eligibility criteria, pilot intervention

Low-acuity ED consulters who declared that they were not attached to a GP practice were eligible to take part in the study, with no restrictions to particular symptoms (both non-traumatic and minor traumatic). Low-acuity was defined as triaged in categories 3–5 of the five-stepped Manchester Triage System (MTS), German version. Additional inclusion criteria were: minimum age of 18 years, ED outpatient treatment, and ability to give formal informed consent (e.g., no substantial language barrier, no mental impairment).

The intervention consisted of two components. In the first instance, an information leaflet about alternative care offers for acute health problems was provided, including examples of constellations and symptoms with corresponding suggestions of adequate care providers: GPs, medical on-call service, EDs, emergency hotlines (see Additional file 1 for an English translation of the text contained in the leaflet). Secondly, all participants were offered to have a personal appointment scheduled with a PC provider. Patients were able to voice their priorities regarding the prospective GP practice, including physician gender, practice size, location, special care offers, and the time of appointment. Study personnel then arranged a suitable appointment at a local practice. Practices were regular GP offices (researched online by study personnel) and did not constitute part of our network. Appointments were requested by phone. There was no strict time limit, but we aimed for scheduling in the two weeks after the ED visit, which was predominantly achieved. In case of out-of-hours ED visits, appointments were arranged when practices were open again, and then relayed to the participants.

### Research network, setting, and recruitment

This pilot study was conducted in the Berlin research network EMANet (Emergency and Acute Medicine Network for Health Care Research Berlin). Patients were recruited in three EDs in central Berlin (Charité – Universitätsmedizin Berlin Campus Mitte and Campus Virchow Klinikum, and the Jewish Hospital Berlin). Recruitment was largely conducted during physicians’ regular office hours, which corresponded to the working hours of the recruiting study personnel. Findings of the qualitative interview study accompanying the intervention have already been reported [11]. Another observational study module of the EMAPREPARE project is focused on the redirection potential of Emergency Medical Services patients.

### Data collection, instruments, variable definitions

Patients were surveyed by study personnel at baseline (= tablet-based interview at the time of the index ED visit, t0 survey) while waiting to be seen. This was conducted after consent and receipt of the information leaflet, but before the optional GP appointment was scheduled. The quantitative questionnaire consisted of 47 items on demographics, medical characteristics (e.g., general health, mental health, chronic conditions), health care utilization (HCU), reasons for not being attached to a GP, subjective urgency, as well as motives for ED consultation. It included validated instruments as well as custom-designed items that had been successfully used in preceding EMANet surveys [8, 12].

Migration history was defined as not being born in Germany and not being a tourist. Education status was scaled according to the CASMIN (Comparative Analysis of Social Mobility in Industrial Nations) classification [13]. Chronic conditions were inquired by a comprehensive multiple-choice checklist ordered by organ systems. For general health, a 5-point Likert scale was used (very good to very bad) [14], and general life satisfaction was measured by the short scale L-1 [15]. For mental health, the PHQ-4 instrument was used (possible values of 0–12 and sub-scores of 0–6 for anxiety and depression) [16], and data on HCU were collected through questions oriented on the German Health Interview and Examination Survey for Adults [17]. HCU at baseline was inquired for a retrospective six-month period. Some questions, e.g., concerning reasons for not being attached to a GP or visiting the ED, were formulated openly, with interviewers matching responses to listed options. Free text documentation was additionally possible.

A FU interview (t1 survey) was conducted by telephone after twelve months; patients could also opt to receive a written questionnaire to be returned by e-mail or letter. The FU questionnaire covered retrospective valuation of the ED visit, satisfaction with the interventional material and services, general and mental health (assessed correspondingly to t0), PC situation, and HCU during the twelve-month FU period. For validation purposes, we additionally contacted the GP practices where patients had appointments scheduled by our study team. These practices completed a short questionnaire in which they indicated whether, when, and how often the respective patient presented there. Data on diagnoses and treatments were not collected from the practices.

### Data preparation and analysis

Most data were used for analysis as collected, but preparation was needed to optimize usability of some variables. For reporting and analysis, the CASMIN education scale was trichotomized into low, intermediate, and high educational attainment. For Likert-scaled data on self-reported general health, data of the upper two categories (very good/good) and lower two categories (bad/very bad) was combined. These data reduction steps were performed prior to the initial analysis on a theoretical basis. Retrospective t0 HCU data were annualized to enable comparability with the FU data, which referred to the whole twelve-month post-ED period [18]. For some questions in the survey, multiple answers were possible (e.g., reasons for not being attached to a GP), thus there is overlap between categories as implicit to multi-response data. Reported ED consultation motives were classified into thematic categories based on the Coster et al. framework [19]. Similarly, thematically related reasons for not having a GP were sorted into summary categories. While questions concerning utilization reasons did not allow for prioritization, they additionally contained an option for open answers. These free text responses were analyzed manually and attributed to existing categories where fitting.

Data were analyzed in SPSS Version 29 (IBM Corp., Armonk, NY, USA) and R 4.1.2 (R Foundation for Statistical Computing, Vienna, Austria). Firstly, descriptive statistics were performed. Groups were compared by Mann-Whitney-U-test for continuous data and χ2 test for categorical data, longitudinal within-subject comparisons were performed with the Wilcoxon signed-rank test. For correlation analysis of binary variables, Pearson’s phi coefficient was calculated. Tentative investigations into potential determinants of accepting the GP appointment service were conducted using binary logistic regression. For all analyses, a significance level of 0.05 was set.

## Results

### Population characteristics, FU yield

Between December 2020 and April 2022, *n* = 317 patients at first screening appeared eligible for the study, based on case characteristics at the ED index visit. Of these, *n* = 157 cases were excluded, with language barriers (*n* = 62), refusal to participate (*n* = 44), and later inpatient admission (*n* = 21) the most common reasons. A total of *n* = 160 patients were eventually included. Of these, *n* = 150 (93.8%) were recruited during usual office hours (08:00–18:00, weekdays). The twelve-month FU was concluded in May 2023 with a total of *n* = 114 respondents. Demographic, health-related and consultation-specific baseline data is summarized in Table 1 for the total study cohort and the subgroup participating in the FU.

**Table 1:** Baseline survey (t0): characteristics of total cohort and patients with FU data available

Variable	Measure	Total cohort	FU sub-cohort
**Participants**	*n*	160	114
***Demographics***			
**Age**	*n*	160	114
	Mean (SD)	30.6 (8.7)	30.9 (8.6)
	Median (Range)	29.0 (18–65)	29.5 (19–65)
**Sex**	*n*	160	114
Male	%	51.2	51.8
Female	%	46.9	46.5
Diverse	%	1.9	1.8
**Migration and travel**	*n*	159	113
Migrant	%	28.9	29.2
Tourist	%	1.3	0.9
**Education (CASMIN)**	*n*	159	113
Low	%	6.9	3.5
Intermediate	%	40.9	41.6
High	%	52.2	54.9
**Living situation**	*n*	157	112
Single household	%	35.7	37.5
**Social situation**	*n*	155	112
Steady partnership	%	55.5	56.3
***ED symptoms***			
**Triage category**	*n*	159	113
3	%	46.5	45.1
4	%	41.6	53.1
5	%	1.9	1.8
**Symptom duration**	*n*	160	114
Started today		15.6	16.7
Started yesterday		25.0	27.2
Several days, but < 1 week		32.5	32.5
> 1 week		26.9	23.7
**Symptom-associated distress**	*n*	157	111
	Mean (SD)	6.4 (1.9)	6.3 (1.9)
	Median (Range)	7.0 (1–10)	7.0 (2–10)
**Subjective urgency**: **I must be seen…**	*n*	156	112
…immediately	%	13.5	8.0
as soon as possible	%	20.5	18.8
today	%	46.8	51.8
not as urgently	%	19.2	21.4
***Health***			
**Morbidity**	*n*	152	109
Any chronic condition	%	15.1	14.7
≥ 2 chronic conditions	%	5.9	5.5
≥ 3 chronic conditions	%	3.3	3.7
**PHQ-4 anxiety subscale**	*n*	159	114
	Mean (SD)	1.7 (1.7)	1.5 (1.5)
	Median (Range)	1.0 (0–6)	1.0 (0–6)
**PHQ-4 depression subscale**	*n*	159	114
	Mean (SD)	1.5 (1.7)	1.5 (1.6)
	Median (Range)	1.0 (0–6)	1.0 (0–6)
**General health**	*n*	160	113
Very good / good	%	83.0	85.8
Average	%	11.3	8.0
Bad / very bad	%	5.7	6.2
**General life satisfaction**	*n*	157	113
	Mean (SD)	7.4 (1.9)	7.6 (1.7)
	Median (Range)	8.0 (0–10)	8.0 (0–10)

Note. *n* = cases with available data for respective characteristic; % = percentage of cases with available data; Ranges reported with median values refer to minimum and maximum; Sex: self-reported; Migration and travel: Migrant = not born in Germany, not a tourist; Education: CASMIN (Comparative Analysis of Social Mobility in Industrial Nations) scale, trichotomized; Triage category: Manchester Triage system (MTS); Subjective symptom-associated distress, general life satisfaction: 0–10 scales; PHQ-4 anxiety and depression: 0–6 subscales; General health: 5-point Likert scale

The study population was characterized by a young age average, few chronically ill patients, and a prevailing self-assessment of general health status as good or very good (> 80%).

About three quarters (72.3%, data for *n* = 159) of the patients currently not attached to a GP practice reported to have had a regular PC provider sometime in the past. However, 40.9% of these participants reported to have had no such attachment for at least the past five years (no GP for 3–5 years: 22.7%, for 1–2 years: 36.4%, data for *n* = 110).

Baseline data of the subgroup which responded to the FU did not indicate any marked differences compared to the overall cohort.

### Motives for ED presentation and lack of GP attachment

As shown in Table 1, most patients were triaged in MTS categories 3 and 4 at the ED, which in the German MTS version corresponds to a targeted time to be seen of 30 min and 90 min, respectively [20]. Symptom onset was more than a day ago in most cases. Subjective symptom-associated distress was 6.4 on an 11-point rating scale, and ~ 20% of the patients believed that their condition was “not as urgent”, equivalent to not needing a same-day evaluation. When asked whether a GP could have solved their acute health problem, 28.3% of the t0 cohort assented (data for *n* = 106).

Motives for the ED consultation were inquired at t0 in a multiple-choice multi-response item. Correspondingly, reasons for not being currently attached to a GP were assessed. Good health and personal mobility were mentioned most frequently here, ‘mobility’ referring to situations associated with a change of residence. Table 2 shows the data for these questions.

**Table 2:** Baseline survey (t0): motives for visiting ED and reasons for GP non-attachment

Motives for ED visit (*n* = 160)	%	Reasons for not being attached to a GP (*n* = 160)	%
**A** Subjective distress and anxiety	70.0	**G** Mobility	53.1
**B** Superiority of the hospital	38.1	**H** Good health	50.0
**C** Access problems in PC	26.9	**I** General preference of specialists	23.1
**D** No evident alternative (“did not know where else to go”)	19.4	**J** Bad experiences in PC	11.3
**E** Convenience	9.4	**K** Former practice has closed	6.9
**F** Mobility (visitors, moved, etc.)	5.6		

Note. Multi-response data with category overlap, thus percentages cannot be added up. Reason groups with ≥ 5% affirmative answers are reported

Seemingly corresponding themes feature in both sets of motivations, but responses are not statistically correlated: Mobility (F and G, overlap in 6 cases, phi 0.07, *p* = 0.402), health-related (A and H, overlap in *n* = 52 cases, phi 0.11, *p* = 0.168), considerations regarding the competence of a provider or care level (B and I, overlap in *n* = 19 cases, phi 0.149, *p* = 0.059), as well as access or organizational barriers in PC (C and K, overlap in *n* = 2 cases, phi 0.05, *p* = 0.5).

### Acceptance and assessment of interventional offers

The offer to optionally have a GP appointment scheduled was assessed as “very good” and “good” by 54.2% and 35.5% of study participants, respectively (5-point Likert scale, data for *n* = 155). However, not all patients who rated the appointment service positively actually opted for a GP appointment: about half of the participants (52.5%, *n* = 84) made use of the scheduling service, and suitable personalized GP appointments could be arranged. In the FU cohort, the share of patients who had accepted the appointment service was 57.0% (*n* = 65). Of these, 73.4% (*n* = 47) reported at t1 to have consequently visited the practice, and more than two thirds were either “very satisfied” (43.5%) or “satisfied” (28.3%) with the appointment they had experienced (data for *n* = 46). At FU, 23.8% of surveyed patients who had made use of the scheduling service still reported no attachment to a GP practice (data for *n* = 63). Of the subgroup that reported visiting the referred practice, only 17.4% still recounted having no GP (data for *n* = 46).

We investigated for potential determinants of accepting the offer of the GP scheduling service. In univariable statistics, there was no significant association with important demographic characteristics (sex, age, education, migration history), a good self-rated general health status, nor with having been attached to a GP in the past. Patients who reported a chronic condition appeared significantly more likely to make use of the service (χ2 test, *p* = 0.027). However, when trying to examine this further in a logistic regression model, we found this association no longer significant when controlling for the central demographics age, sex, and education (*p* = 0.078, OR 2.65, 95% CI [0.90; 7.83]). Altogether, with the available data, we could not build a meaningful logistic model for service acceptance as outcome.

Concerning the educational part of the intervention, most participants assessed the information leaflet as “very good” (16.9%) or “good” (62.5%) (5-point Likert scale, data for *n* = 142), and 92.9% were affirmative to the concept of providing information on contact points for acute care options in the situational circumstances of an ED visit. However, at FU only 5.4% of the participants said they had used the information again in a situation with urgent health concerns (data for *n* = 112).

### FU survey: trends in self-reported general and mental health

Longitudinal trends in self-rated general health and general life satisfaction could not be observed, however PHQ-4 mental health scores decreased from t0 to t1, indicating a reduction of mental symptom burden (Table 3).

**Table 3:** Longitudinal comparison for general health, general life satisfaction, and mental health

Variable	Total cohort, Mean (SD) t0	Cases with t0 and t1 data, Mean (SD) t0	Cases with t0 and t1 data, Mean (SD) t1	*n* cases with t0 and t1 data	*p* for difference
**General life satisfaction**	7.4 (1.9)	7.6 (1.7)	7.7 (1.7)	111	0.467
**General health**	1.9 (0.9)	1.8 (0.8)	1.9 (0.8)	113	0.090
**Mental health (PHQ-4)**					
Total	3.2 (3.0)	3.0 (2.7)	2.3 (2.7)	114	0.003
Anxiety subscale	1.7 (1.7)	1.5 (1.5)	1.2 (1.5)	114	0.032
Depression subscale	1.5 (1.7)	1.5 (1.6)	1.1 (1.5)	114	0.016

Note. *p* for difference: Wilcoxon signed-rank test; General life satisfaction: short scale L1 (0–10); General health: 5-point Likert scale (1-5, 1 = ‘very good’); PHQ-4: 0–12 scale; PHQ-4 anxiety and depression: 0–6 subscales

### FU survey: trends in self-reported HCU

**Fig. 1:**
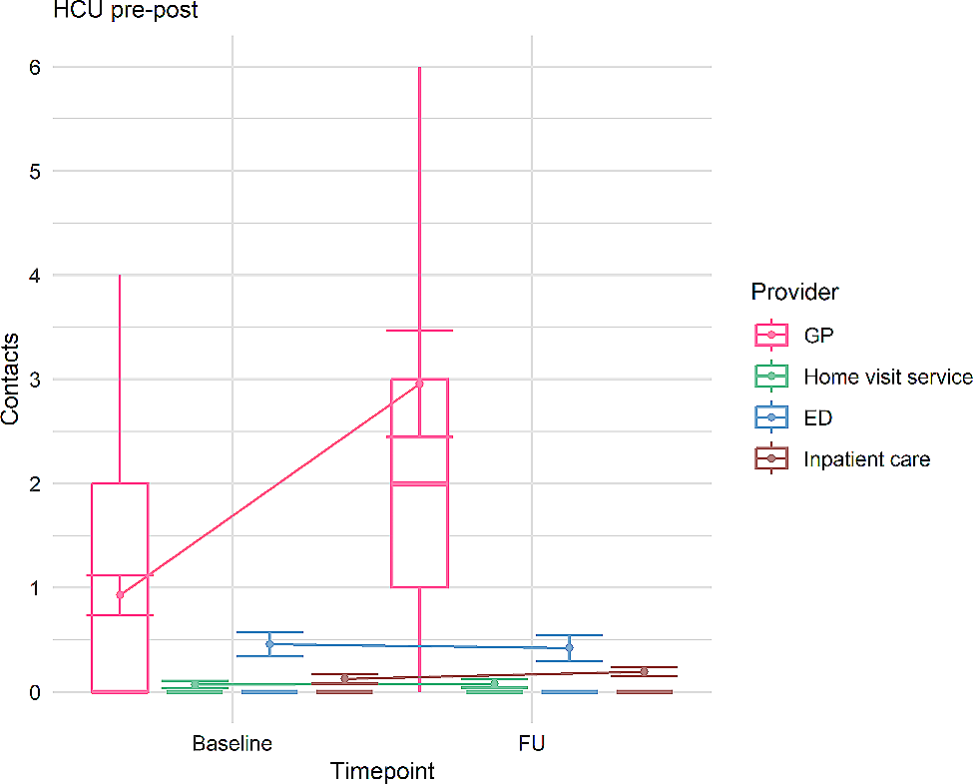
Longitudinal trends in HCU. *Note*: Retrospective self-reported annualized HCU for survey time points t0 and t1. Points show means, error bars represent standard errors of means. Boxes correspond to medians / quartiles, whiskers to the largest observed point falling within distance of 1.5 interquartile range. As median consultation frequency is zero for all providers except GPs, no boxes are graphed for these. Line chart elements represent longitudinal trends

HCU surveys showed a marked and statistically significant increase in GP utilization in the FU cohort compared to baseline HCU, with the contact rate essentially tripling. For other providers of acute care (use of home visit service, ED visits) and for hospital inpatient care, no longitudinal differences in utilization could be found (Table 4; Fig. 1).

**Table 4:** Longitudinal comparison of HCU data

Provider	Total cohort: contacts pre t0, Mean (SD)	Cases with t0 and t1 data: contacts pre t0, Mean (SD)	Cases with t0 and t1 data: contacts pre t1, Mean (SD)	Utilization rate during FU period, %	*n* cases with t0 and t1 data	*p* for difference
**GP**	0.9 (1.9)	0.9 (2.0)	3.0 (5.5)	82.3	111	< 0.001
**Urgent care home visit service**	0.1 (0.4)	0.1 (0.4)	0.1 (0.4)	4.4	114	0.952
**ED**	0.6 (1.3)	0.5 (1.2)	0.4 (1.3)	20.2	114	0.462
**Hospital inpatient care**	0.2 (0.6)	0.1 (0.5)	0.2 (0.4)	17.5	112	0.504

Note. *p* for difference: Wilcoxon signed-rank test; Contacts prior to t0/t1: annualized utilization data; If a patient stated to have visited a provider, but did not report how frequently, visit count was set as = 1

Patients with FU data available who did not make use of the GP appointment service had a mean number of 1.5 GP consultations during the FU period (SD 1.6, median 1.0, range 0–6). In contrast, patients who made use of the service had an average consultation frequency of 4.1 (SD 6.9, median 3.0, range 0–52) (*p* < 0.001, Mann-Whitney-U-test, data for *n* = 113 cases). No difference between the two groups could be observed with regard to ED consultations in the FU period (ED visits in patients who did not use the appointment service: mean 0.3, SD 0.9, median 0.0, range 0–5; ED visits in patients who did use the appointment service: mean 0.5, SD 1.6, median 0.0, range 0–12; *p* = 0.198, Mann-Whitney-U-test, *n* = 114 cases). Neither for participants of the appointment scheduling service, nor for non-participants, did average frequency of ED contacts in the time prior to the index visit differ significantly from the follow-up period.

### Practice survey: validation of PC utilization take-up

The practice survey verified an appointment take-up rate of 63.0% (*n* = 51) in the overall population of patients who made use of the scheduling service (data for *n* = 81). For patients who had visited the practice in which an appointment had been scheduled via EMAPREPARE, practices reported a mean number of 3.1 (SD 2.2) consultations with personal physician contact over twelve months (data for *n* = 51). In case of patients who themselves stated that they had visited the practice where they had an appointment arranged, this could be verified in the practice survey in 87.0% of cases (40 cases out of *n* = 46 with available data).

## Discussion

### Summary of findings

This pilot study focused on patients not connected to a GP and investigated the feasibility and acceptance, as well as some indicators of potential effects, of an ED-based intervention combining written information on acute care options with a personalized PC appointment scheduling service. The included *n* = 160 patients were characterized by comparatively young age, high level of education, and lack of chronic disease. Reasons for not being attached to a GP most prominently comprised good health, personal mobility, preference for specialists and bad experiences with PC. ED consultations in our cohort seemed frequently triggered by subjective distress and anxiety. Additional determinants included a belief in the superiority of the hospital and access problems in PC. The intervention services were positively evaluated, and more than half of the participants accepted the scheduling service offer. In our study data, we could however not identify any potential determinants for the acceptance of this interventional component. Three quarters of service users reported attending the practice in which they had received an appointment, and the validity of self-report could be essentially verified in the practice survey. FU data suggested a tripling of GP utilization compared to baseline. The practice survey indicated comparable PC consultation frequencies. There was no significant change in utilization of other healthcare services, including ED visits.

### Results in context

#### Determinants and consequences of ED utilization, PC attachment and care continuity

The population of low-acuity ED patients without GP attachment differs markedly from a general ED population in their lower average age [20], lower likelihood of chronic disease and comparably high education level [1]. Consistent with our findings, many studies have characterized low-acuity presenters as younger than unselected ED patients [21–23]. Subjective distress and anxiety concerning their current medical symptoms represented by far the most common motive for preferring the ED over PC options. Concurrently, about 80% categorized their symptoms as urgent, equivalent to the need for a same-day evaluation. In line with this, subjective distress and urgency have been reported as major motives for low-acuity ED utilization [24, 25], but then again as a poor indicator of appropriateness [26]. Access barriers in PC constitute another main reason for low-acuity ED self-referrals and have been reported from many settings [10, 27]. This issue was mentioned by a quarter of our study population, corresponding in scale to e.g., the findings of Brasseur et al. in a Belgian care context [28].

Concerning reasons for not being attached to a GP, personal mobility factors and the assessment of their own health status as good were most prominently mentioned in our study. Mobility – e.g., frequent changes of place of residence for reasons of work, study etc. – in turn is associated with the lifestyle of younger populations and may itself be considered an access barrier to PC, as it hinders continuity of care.

Altogether, our population seems to reflect a specific type of healthcare utilizer which is distinct from most ED patients, but remarkable in the context of low-acuity utilization. While only few studies have investigated characteristics of patients without a GP, our findings are generally in line with the determinants described in a large population-based German study [9]. However, their decision-making is certainly complex, as our investigation into the interrelations of motives for ED use and reasons for not being attached to a GP indicates, with prima facie similar motives not relevantly correlated. Our qualitative findings concerning the intervention reflect this complexity [11].

For this special population however, which has comparatively sporadic contact with the health care system, ED consultations could represent a suitable window of opportunity for interventional efforts to modify consultation patterns: patients could be responsive to such interventions in a situation of concern for their own health. Similar processes have been discussed in the context of substance use disorder and other mental health issues [29, 30].

#### Efforts to promote PC attachment: educational and organizational

While the interventional offers were altogether favorably valued, we can only speculate why a substantial share of patients still rejected the appointment scheduling service, since statistical investigations into potential predictors for acceptance vs. dismissal did not yield any clues. We must thus concede that the variables collected in our study do not allow a suitable explanation, suggesting that the roots of decision-making are complex and multifaceted, with potentially relevant factors such as previous care experiences and underlying views probably being best captured by qualitative research [11, 31, 32].

The educational component of our intervention was theoretically based on findings of health literacy being associated with both ED utilization and access problems in PC [10, 23, 33–35]. Regarding information provision, de Steenwinkel et al. and Minderhout et al., have stressed the importance of target group-specific informational measures for promoting health literacy about ED usage [10, 36]. In the de Steenwinkel study, patients preferred oral information and leaflets over other forms of information provision [36]. Other authors however have raised concerns regarding the effectiveness of standalone educational information for influencing ED utilization and have advocated for its inclusion in multifaceted interventions [37]. Considering our results, we must concede that – while it was well-received – the leaflet was unlikely to have had an impact, as indicated by the low re-use rate of the information reported in the FU survey. Furthermore, it is difficult to separate potential effects of information provision from those of the add-on appointment service, as all participants received the flyer. Speculatively, the appointment service component’s effects on future utilization decisions could be less volatile compared to written information, as it is associated with higher commitment and lower transiency. This is evidenced by the high take-up of appointments, adherence indicating that this is not just a situational flash in the pan. The confirmation of validity through the practice survey additionally eliminates concerns about social desirability that may be associated with the HCU inquiries. Consistent with our findings, organizational PC-centered interventions were frequently found effective for modifying utilization, but systematic evidence syntheses are far from conclusive in terms of best practice due to the heterogeneity of approaches studied [38]. PC appointment frequencies – as seen in the patient and practice surveys – also suggest some degree of care continuity beyond the initial arranged visit. This interventional offer thus appears to be helpful in overcoming a potential obstacle to PC attachment: the hassle of scheduling appointments, especially if e.g., one is new in the city or neighborhood [39, 40]. Web-based systems for self-scheduling of appointments could also be a suitable solution to reduce such access barriers [41].

From a theoretical perspective, a regular GP-based utilization pattern is commendable, as care continuity is associated with benefits to health-related outcomes [42, 43]. However, from an economic viewpoint and in consideration of ED crowding, it would be best if the increased utilization of PC were accompanied by a decrease in ED use, instead of representing additional utilization that could be seen as a supply-induced demand effect [44]. We could not detect such decreases in our data; however, this could be due to smaller effects not being visible in a limited-scale pilot trial. Consequently, we must emphasize the importance of investigating this further in larger controlled studies. For countering conceivable supply-induced demand effects created by more convenient ambulatory care structures, restrictions to free ED access could be discussed (e.g., penalty co-payments for low-acuity cases), but such measures are highly controversial as to their potentially detrimental effects on equity in accessing health care [45, 46]. In contrast, a PC-based health care system with mandatory registration at a GP practice and restricted direct access to specialist and hospital healthcare could constitute a solution for increasing GP utilization without negative impact for vulnerable populations [47, 48].

### Limitations

The small sample size of this pilot study must certainly be stressed as its most important limitation, as well as the non-controlled before-after design. Concerning sample size, the limited recruitment potential is partly due to the inclusion criterion ‘not attached to a GP’, which only applies to a small proportion of the patient population. However, the seemingly long recruitment period necessary also had organizational reasons, primarily the pandemic situation with periods of general suspension of recruitment at our institution, as well as personnel shortages. The pandemic is also likely to have impacted utilization decisions and patterns in both EDs and PC, so we cannot say whether the results (e.g., HCU frequencies) would have been similar in non-pandemic times. In a large national study, around 10% of respondents reported forgoing a medical consultation due to COVID-19, with the impact on GP utilization comparably greater than on ED visits [49]. As mentioned earlier, the limited population size and aggravating FU attrition may also have hindered the detection of potentially important effects, as the study was underpowered in this respect. Furthermore, quantitative surveys may be ill suited to capture the complexity of utilization motives and decision-making processes, which is why our study used complimentary qualitative methods. It is also possible that our results reflect some specific features of the German health care system, with e.g., GP attachment not being formalized, as well as the metropolitan setting of central Berlin, with its comparably mobile population and broad spectrum of health care providers.

## Conclusions

This pilot study suggests that a personalized appointment scheduling service is a promising approach to promote PC attachment among patients without a GP, with longitudinal data showing marked increases in PC utilization. However, effects on ED usage are uncertain and require further investigation in larger-scale controlled studies.

### Electronic supplementary material

Below is the link to the electronic supplementary material.

Additional file 1. Text of the information leaflet provided in the EMAPREPARE intervention [translation to English language].

## Data Availability

The datasets used and analyzed during this study are available from the corresponding author on reasonable request.

## Abbreviations

ED: Emergency department
PC: Primary care
GP: General practitioner
EMAPREPARE: Emergency and Acute Medicine - Primary Care demands in patients resorting to emergency departments
FU: Follow-up
MTS: Manchester Triage System
EMANet: Emergency and Acute Medicine Network for Health Care Research Berlin
HCU: Health care utilization
CASMIN: Comparative Analysis of Social Mobility in Industrial Nations
PHQ: Patient Health Questionnaire
SD: Standard deviation
COVID-19: Coronavirus Disease 2019
