# Supplementary material for: Emergency department visits as a potential opportunity to promote primary care attachment and modify utilization patterns – results of a pilot study in Berlin, Germany
Source: BMC Emerg Med. 2024 Aug 7;24:142. doi: 10.1186/s12873-024-01056-0 (PMC11304799; doi:10.1186/s12873-024-01056-0)
Supplement: Supplementary file 1 — Additional file 1. Text of the information leaflet provided in the EMAPREPARE intervention [translation to English language]. [file 12873_2024_1056_MOESM1_ESM.pdf]

Emergency department visits as a potential opportunity to promote primary care attachment and modify utilization patterns – results of a pilot study in Berlin, Germany

Felix Holzinger, Lisa Kümpel, Rebecca Resendiz Cantu, Anja Alberter, Martin Möckel, Christoph Heintze

---

Additional file 1 – Text of the information leaflet provided in the EMAPREPARE intervention [translation to English language]

Emergency – what now?

A little guide to help you decide.

<u>General practitioner (GP)</u>

In the event of illness, a general practitioner should normally be the first point of contact. They not only treat chronic illnesses, such as high blood pressure, or carry out annual check-ups, but also look after for patients with acute complaints.

For example, you should see a GP if you have the following symptoms and concerns:

• Rhinitis and cough

• Sore throat and hoarseness

• Digestive problems and diarrhea

• Headache and back pain

• Bladder infections

• General health advice

Many GPs also offer short-term appointments so that complaints can be investigated promptly. If necessary, a GP can also refer you to specialist care.

Appointment service:

If you need help to get an appointment with a GP, specialist, or psychotherapist quickly, you can contact the appointment service centers of the Association of Statutory Health Insurance Physicians (KV). Further information can be found on the KV Berlin website www.kvberlin.de (patient section), or by calling 116 117 (free nationwide).

<u>Medical on-call service</u>

You can contact the medical on-call service if GPs are not available outside of regular opening hours, e.g. in the evening or at the weekends, and if you have complaints that cannot wait until the next working day.

Possible symptoms are:

• Persistent vomiting and diarrhea

• High fever

• Acute abdominal pain

You can reach the medical on-call service nationwide and around the clock on the free 116 117 hotline.

The medical on-call service provides advice by phone, and, in urgent cases you can make use the home visit service. If necessary, you will also obtain information about urgent care practices in your area.

Emergency department

In the event of potentially life-threatening situations and accidents, you should go to an emergency department immediately or call the emergency number (112) to receive appropriate care in a hospital within a short time.

Important symptoms can be, for example:

• Impaired consciousness

• Acute shortness of breath

• Paralysis

• Severe injuries and bleeding

• Acute chest pain
